# Junior doctors’ experiences of managing patients with medically unexplained symptoms: a qualitative study

**DOI:** 10.1136/bmjopen-2015-009593

**Published:** 2015-12-01

**Authors:** Katherine Yon, Sarah Nettleton, Kate Walters, Kethakie Lamahewa, Marta Buszewicz

**Affiliations:** 1 Research Department of Primary Care & Population Health, UCL, London, UK; 2 Department of Sociology, University of York, York, UK

**Keywords:** MEDICAL EDUCATION & TRAINING, PRIMARY CARE, MENTAL HEALTH, MEDICALLY UNEXPLAINED SYMPTOMS, JUNIOR DOCTORS

## Abstract

**Objectives:**

To explore junior doctors’ knowledge about and experiences of managing patients with medically unexplained symptoms (MUS) and to seek their recommendations for improved future training on this important topic about which they currently receive little education.

**Design:**

Qualitative study using in-depth interviews analysed using the framework method.

**Setting:**

Participants were recruited from three North Thames London hospitals within the UK.

**Participants:**

Twenty-two junior doctors undertaking the UK foundation two-year training programme (FY1/FY2).

**Results:**

The junior doctors interviewed identified a significant gap in their training on the topic of MUS, particularly in relation to their awareness of the topic, the appropriate level of investigations, possible psychological comorbidities, the formulation of suitable explanations for patients’ symptoms and longer term management strategies. Many junior doctors expressed feelings of anxiety, frustration and a self-perceived lack of competency in this area, and spoke of over-investigating patients or avoiding patient contact altogether due to the challenging nature of MUS and a difficulty in managing the accompanying uncertainty. They also identified the negative attitudes of some senior clinicians and potential role models towards patients with MUS as a factor contributing to their own attitudes and management choices. Most reported a need for more training during the foundation years, and recommended interactive case-based group discussions with a focus on providing meaningful explanations to patients for their symptoms.

**Conclusions:**

There is an urgent need to improve postgraduate training about the topics of MUS and avoiding over-investigation, as current training does not equip junior doctors with the necessary knowledge and skills to effectively and confidently manage patients in these areas. Training needs to focus on practical skill development to increase clinical knowledge in areas such as delivering suitable explanations, and to incorporate individual management strategies to help junior doctors tolerate the uncertainty associated with MUS.

**Strengths and limitations of this study**

- This is the first known study to explore newly-qualified doctors’ experiences of managing patients with medically unexplained symptoms (MUS) and to identify postgraduate training needs in this area.
- Our study highlights an important gap in junior doctors’ knowledge about MUS and emphasises the importance of educating doctors at an early and clinically-relevant stage of their career.
- Junior doctors were forthcoming when discussing negative viewpoints towards patients with unexplained symptoms and the challenges and difficulties they have faced.
- We obtained a range of views by ensuring maximum diversity according to gender, age, ethnicity and training level.
- Participants were recruited from the North Thames London region, and the views expressed may not be representative of other newly-qualified doctors within the UK or elsewhere.

## Introduction

A range of studies suggest that 40–50% of cases seen in primary care and around half of new referrals to secondary care can be described as dealing with medically unexplained symptoms (MUS) which are not linked to clear diagnoses of organic pathology.^1–3^ The term MUS encompasses a wide range of presentations and can affect all bodily systems, hence the high number of patients seen across all specialties. The increased rates of presentation, unnecessary investigations and referrals associated with MUS are accompanied by high medical, social and indirect costs.^4–8^ For example, a UK study found the use and cost of medical investigations for frequent users of secondary care with no clear physical diagnosis was significantly greater than for frequent attenders with a diagnosis.^9^ There is currently a strong drive within healthcare systems to reduce costs and therefore a need to educate doctors about appropriate levels of investigation and suitable management strategies for patients with MUS.

A common assumption is that patients with unexplained symptoms pressurise doctors into unnecessary investigations in their search for diagnoses and medical treatments. However, detailed work in primary care settings in the UK has indicated that many patients consulting their general practitioners (GPs) with MUS seek emotional support, explanations and reassurance more than do patients with more straightforward physical diagnoses,^10^ and that some patients come to consultations having already considered a psychological cause for their symptoms.^11^ ^12^ Research with GPs suggests doctors have difficulty eliciting patients’ views about these psychological causes, and feel uncertain and insecure about their ability to deal with patients’ need for emotional support.^13^ ^14^ They often suggest disease-related investigations or treatments that are costly and risk causing iatrogenic harm.^15^ Other research suggests GPs may underestimate their own psychological expertise and ability to explore psychosocial issues with patients, and that this is an important area to address within a training intervention.^16^

Understanding how to manage patients while avoiding over-investigation but not missing significant pathology, and providing explanations which empower patients and enable them to increase the control they have over their symptoms should be part of any clinician's repertoire.^17^ A recent study examining third and fourth year medical students’ attitudes towards MUS found many students had negative views about the causes and management of such presentations, and considered that their current medical training fails to equip doctors to engage with this topic.^18^ Students expressed a wish for evidence-based training at a clinically relevant time and for awareness to be raised before negative attitudes develop;^18^ however surveys have demonstrated teaching on this topic at undergraduate level is sparse.^19^ ^20^

To the best of our knowledge, there is no research to date investigating newly-qualified doctors’ experiences of managing patients with MUS or their related training needs. This study aimed to explore junior doctors’ familiarity with MUS, to identify any gaps in their knowledge regarding management strategies, and to explore their views and recommendations for postgraduate teaching about MUS.

## Methods

### Design

In-depth interviews were used to explore junior doctors’ views and experiences of managing patients with MUS, and their recommendations for future training. This approach was chosen to allow individuals sufficient space to describe their own ideas in detail, and to enable participants to present views which they might not feel comfortable expressing in other settings such as focus groups.

### Participant selection and recruitment

Newly-qualified doctors undertaking the 2-year UK Foundation Training Programme (FY1/FY2) were recruited from three North Thames London hospitals. Doctors were initially introduced to the study via email and the research associate (KY) attended one foundation year teaching session at each of the three hospitals to describe the study, answer questions and collect contact details of those willing to participate. Following this, interested participants were asked to give demographic details and purposive sampling took place to ensure maximum diversity according to gender, age, ethnicity and training level.

### Conducting the interviews

Interviews took place at the hospital sites at times convenient to the interviewees and were conducted by the research associate trained in qualitative interviewing. Four pilot interviews were conducted with a sample of junior doctors and qualified GPs. The topic guide was developed with reference to the background literature and an unpublished qualitative study with GP trainees (personal communication, Howman, 2014) and revisited throughout the interview process to adapt to newly emerging topics (see online supplementary file). The interviews lasted 36 min on average (ranging from 19 to 59 min) and a non-judgemental, exploratory approach was adopted. Questions asked related to junior doctors’ previous experiences of treating patients with MUS, their views about working with such patients, ideas for short and long-term patient management and recommendations for future training on this topic. The interviews also explored their emotional reactions to working with patients with unexplained symptoms and any barriers they perceived to delivery of good care. Data saturation was achieved when the interviews no longer provided new information.

Participants gave written consent prior to participation, and were offered a £20 book voucher to compensate for their time.

### Data analysis

Interviews were audio recorded and data managed using Microsoft Excel. The framework method was selected due to its systematic and rigorous approach to qualitative data management and analysis.^21^ Organising the data in this manner allowed for flexibility and facilitated the process of working collaboratively through a detailed data set in a transparent and organised way. The five coauthors acted as independent reviewers in the systematic data organisation and theme identification stages of analysis. The reviewers brought a multidisciplinary approach to the study, interpreting the data from the varying perspectives of medical practitioners (MB, KW), sociologists (SN, KL) and a psychologist (KY) with educational experience in the field of MUS (MB, SN, KW). Initially, each transcript was closely read by at least two reviewers and data were organised into agreed framework matrices by the research associate. These were then read by all five members of the research team, with the aim of encouraging familiarisation with the data set and to identify emergent and salient issues for discussion. Individual ideas were brought to group discussions, and categories and overarching themes agreed on. Themes were revisited and refined and possible explanations for associations between aspects of the data set were discussed in subsequent team meetings.

Preconceptions and ideas were challenged by other team members at all stages in order to encourage a reflective and thoughtful approach to data analysis. For example, the reviewers were careful to note that their prior experiences of research and teaching delivery in this area might lead them to unconsciously pay more attention to aspects of the data which conformed to existing expectations. Hypotheses such as the idea that junior doctors would struggle with the management of patients with MUS, or that junior doctors would be keen for further training in this under-represented topic were discussed within the team, and care was taken to search for and report on all aspects of the data that disproved as well as approved these hypotheses.

The results have been reported in accordance with the Consolidated Criteria for Reporting Qualitative Research (COREQ) checklist.^22^

## Results

### Sample characteristics

Twenty-two junior doctors working across three hospitals in the North Thames region, who had qualified from 13 UK Medical Schools were interviewed.

Participant demographics regarding training level, gender, ethnicity and age are displayed in table 1.

**Table 1:** Participant demographics

Variable	n/22
Level	
FY1	7
FY2	15
Gender	
Male	9
Female	13
Ethnicity	
White British	14
Other British	5
White Other	1
Other	2
Age (years)	
20–29	14
30–39	7
40–49	1

FY1/FY2, foundation year 1 and 2.

### Attitudes and perceptions towards patients with MUS

#### Role and responsibility

Very few participants reported receiving formal training on the topic of MUS, but they had all encountered patients with unexplained symptoms through their clinical work. Junior doctors described feeling unprepared to deal with such patients, and appeared unsure what they as doctors could offer in terms of on-going management. Some spoke of avoiding communication with such patients altogether due to an uncertainty around how to deliver explanations or discuss appropriate treatment options.

That's the thing I find about medically unexplained symptoms, I've got no answer and I often feel very powerless. …I just don't know what to do. … I've never had to [give explanations], I've deferred, and I probably will keep deferring until I'm the consultant. Hopefully by then I'll have some common skills training to know how to do it. (P12, Male, FY1)

Despite this feeling of helplessness, many junior doctors said they would like to be able to provide more support to such patients, but appeared unsure what suitable management approaches might look like or whether this would be feasible in practice. Several said they would ideally like to be able to sit and talk with patients for longer and thoroughly explore their symptoms, personal and social circumstances and any other contributing factors. Beyond this, there was little consensus about the doctor's role in on-going management, with a sense of therapeutic nihilism in some responses.

As a junior doctor, I can't really do much. (P10, Female, FY2)

Some felt managing patients with MUS was not part of their role, and doubted whether providing care for them was appropriate within the remit of medical services. A few junior doctors thought the management of such patients should fall more within the role of GPs, psychiatrists or psychologists, and some felt their time was best spent dealing with patients with symptoms of a clearly organic nature.

There's so much to learn about stuff that actually you can see and prove goes wrong, …so when you come across something that doesn't fit that dogma you just think well, it's not my job and it's not really my business to be involved in it….So I think it would be helpful in psychiatry training and GP training (P24, Male, FY1).

#### Subtypes of patients

When describing patients with MUS, doctors commonly defined patients as falling within three main subtypes. The first were individuals experiencing symptoms linked to psychiatric or psychosocial factors such as mental health problems, trauma or stress. The second subtype described patients with symptoms linked with organic pathology which had not yet been identified. The final subtype involved patients described as malingering individuals presenting with ‘made up’ symptoms in order to further personal gain.

Some patients… are making it all up. Some patients, this is all in their mind. (P18, Female, FY2)

Descriptions of patients experiencing symptoms linked to physiological causes of a non-organic or non-pathological nature were seldom discussed. Instead, junior doctors indicated a need to find an adequate explanation for patients’ symptoms, whether this be in relation to organic disease or a psychosocial explanation. Regardless of the supposed origins of patients’ symptoms, many doctors acknowledged the difficulties faced from the patient's perspective.

Whether [the symptoms] are really actually there, or whether it's a manifestation of some kind of psychiatric disorder or something else, I do think that generally people do suffer from them” (P24, Male, FY1).

#### Current management and use of investigations

Many junior doctors felt that both they and other health professionals tended to over-investigate patients, particularly where the diagnosis was unclear. For many, the fear of missing something serious and facing negative consequences was identified as a factor contributing to over-investigation.

There is a lingering fear of missing something or not diagnosing something. Wanting to avoid litigation can sometimes be a driving factor pushing you towards doing lots of investigations. (P07, Male, FY2)

Some mentioned that investigating patients was a more appealing option, used to minimise time spent talking to patients and to avoid having to construct and deliver explanations for ambiguous symptoms.

I think it's all too easy when somebody like that comes in just to do the investigation so you can get rid of them a bit quicker. It takes a lot longer to try and talk to them about it. (P15, Male, FY2)

A small number were more confident in their approach, and able to put into practice specific management strategies they had developed themselves, or in some cases had learnt through brief training.

I think you have to offer bespoke explanations depending on the person. … So what you would tend to say is ‘occasionally these types of symptoms we can't explain, despite being completely real to yourself, and they can occasionally cause people to feel very anxious and low in mood’ (P05, Male, FY2).

### Barriers to effective management

#### Organisational constraints

Patients with unexplained symptoms were often described as individuals demanding a lot of attention, and many doctors felt they did not have enough time or resources to be able to meet their needs. In some cases, patients with MUS were not considered as worthy of doctors’ time as patients experiencing disease-related symptoms, particularly in busy settings such as inpatient wards or A&E.

There were other people who were more sick, and other things to do and organise (P10, Female, FY2)

Pressures to make swift decisions and attend to patients within allocated time frames also meant doctors took less time to explore the psychosocial aspects of symptoms or deliver explanations, and instead preferred to refer patients back to their GPs. Despite this, many doctors recognised the gaps in service delivery and suggested they would prefer to be able to spend more time exploring patients’ needs.

Having a longer appointment with her would be really useful, just one hour …to put together a nice summary of what the symptoms are, all the investigations and their results, and then decide what to do next, rather than just passing her from service to service. (P21, Female, FY2).

#### Psychological constraints

Patients with unexplained symptoms often appeared to trigger negative feelings in the junior doctors such as annoyance, frustration, confusion and anxiety.

They're frustrating, because there's nothing you can do to help them (P16, Female, FY2)

This apprehension appeared to affect doctors’ willingness to work with such patients, and also their confidence in their ability to manage cases or provide effective support. The uncertainty associated with MUS seemed linked to a feeling of incompetence, particularly as they were more accustomed to dealing with cases involving clear organic pathology.

I much prefer dealing with problems where I know the cause for the problem or at least I know how to go about finding a cause. (P07, Male, FY2)

Some junior doctors indicated that they avoided suggesting a psychosocial connection to symptoms, fearing this might offend patients or leave them feeling as though they had not been believed or taken seriously. The uncertainty around both the cause of the symptoms and possible physiological or psychological explanations left the doctors feeling unsure how to communicate with patients when discussing test results or management ideas.

She said the classic line, ‘are you telling me it's all in my head doctor?’ And whilst we thought it might be something to do with that, it was a really difficult subject to broach with her. (P12, Male, FY1).

#### Role modelling as the main source of training

A lack of formal training in the topic was described by most respondents. Participants indicated that the management techniques they had adopted were often a result of the informal observation of others. Exposure to negative views of other staff members, particularly those more senior, was thought to have influenced their attitudes and treatment of such patients, as patients with MUS might at times be described as ‘crazy’ or ‘mad’ by seniors and recommended for quick discharge. Junior doctors considered that ‘going against the norm’ and approaching cases differently would not be met positively by their seniors. Given their lack of status and power, juniors often felt less confident in suggesting alternative ways of managing such patients, such as spending more time exploring their difficulties.

Some people [seniors] would sort of scoff at it, and you know, just be derogatory about it. (P17, Female, FY1).

### Recommendations for training

#### Views about training

Junior doctors recognised the significant impact of MUS on NHS costs, resources and professionals’ time. They thought that raising awareness about how common MUS are and providing more training about how to manage patients would be helpful considering the high numbers of such patients encountered and their lack of knowledge about clear and appropriate management strategies. A few participants thought that training about the management of MUS would be more relevant to the training of GPs and psychiatrists, as they thought that doctors working in those fields would encounter more of these patients. Very few junior doctors had received any teaching on this topic during medical school, and most thought relevant training would help them to feel better prepared when considering how to approach cases in inpatient and outpatient settings.

Given that MUS is very common, and actually takes up a heck a lot of resources, I think it's a good idea [to provide training]. (P18, Female, FY2).

#### Content of training

Case-based discussions and practical communication skills sessions were recommended as appropriate teaching methods. Those interviewed thought that discussing ideas in a group setting would be a useful way to share ideas and consider the experiences of both senior and junior doctors. Other approaches mentioned were problem-based learning (PBL), the use of videos and role play with peers or simulated patients, although views on role play were mixed. Lecture-based teaching was not recommended as it was considered that the topic of MUS requires an interactive approach.

I think videos can be quite useful, because then you can relate what you’re learning to an actual physical patient. …it sticks in your head. (P20, Female, FY1)

Participants spoke of the need to raise awareness about MUS during undergraduate teaching, with more formalised teaching provided at times of greater clinical exposure such as during the foundation years or later during core medical training. They suggested that training should focus on communication techniques, particularly in relation to the communication of negative test results and specific examples of delivering physiological and psychological explanations for symptoms.

Having a satisfactory explanation to give a patient, and expressing the uncertainty I've got without necessarily rubbing them up the wrong way would certainly be a good start (P12, Male, FY1)

As well as focusing on effective communication, there was a request for more information about what doctors could provide for people with unexplained symptoms in terms of short and longer term management strategies. Many doctors reported feeling helpless and unsure about their role in patient management, and thought information about appropriate referral options, community-based support and psychological services would be helpful when considering longer term management.

## Discussion

This qualitative exploration of junior doctors’ experiences of managing patients with MUS highlights a large gap in their knowledge about the topic and appropriate patient management, and emphasises the urgent need for improved postgraduate teaching for newly-qualified doctors.

Junior doctors described patients with unexplained symptoms as a challenging group of individuals who are often perceived as ‘impossible to help’, and some questioned the legitimacy of such patients’ demands on doctors’ time and resources. Similar attitudes towards patients with unexplained symptoms have been reported in other studies with GPs in primary care settings,^14^ ^23^ ^24^ and are thought to stand in the way of doctors’ ability to provide optimal care.^25^ ^26^ Some of the negative views that junior doctors held towards patients with MUS appeared linked with their exposure to the dismissive attitudes of senior role models; findings similar to that of a recent study examining the attitudes of medical students.^18^ These emphasise the need to raise awareness about MUS during the early stages of doctors’ careers, ideally before negative attitudes are formed and to address issues relating to the attitudes of seniors within training posts.^27^ Junior doctors indicated that receiving more in-depth and clinically relevant training during their foundation years about ways in which they could provide effective practical and emotional support to such patients would be helpful. They emphasised the need for training to be interactive and based on their own case experiences in order to encourage active group discussion.

Junior doctors appeared unclear about their role in patient management, and spoke about avoiding conversations or ordering multiple tests because of this uncertainty. In some cases, investigations seemed to be used as an avoidance strategy to defer dealing with patients’ worries, and were also instigated to avoid the possibility of litigation. Feelings of incompetency and discomfort in relation to MUS have been reported by doctors at all career stages^28^ ^29^ and may lead to the adoption of unhelpful approaches that could leave patients feeling unheard and as though they have not been taken seriously.^30^ Reattendance rates may also increase as patients search for adequate explanations for their symptoms and definitive diagnoses.^4^ ^9^ Ordering investigations in order to reassure the doctor rather than the patient is thought to be a common yet unhelpful strategy,^10^ and is both costly and risks iatrogenic harm.^15^ It is important for any training intervention to educate doctors about appropriate levels of investigation and to encourage, where indicated, the effective use of appropriate strategies which have been found helpful, such as providing reassurance, demonstrating empathy and giving effective explanations for symptoms.^10^ ^30^ ^31^ Assisting doctors to separate feelings of uncertainty from a feeling of incompetence is crucial, as is reinforcing the fact that doctors of all levels find the management of patients with MUS challenging due to the nature of dealing with ambiguous symptomology.

Improving doctors’ understanding of the mind and body as integrated as opposed to separated entities may help to address the need to find organic causes for patients’ symptoms and encourage a more holistic approach to management. Providing relevant examples during training of both psychological and physiological explanations for unexplained symptoms could have the potential to improve doctors’ confidence when approaching difficult topics or delivering negative test results.

### Strengths and limitations

To the authors’ knowledge, this is the first study to explore newly-qualified doctors’ experiences of managing patients with MUS and to identify postgraduate training needs in this area. Junior doctors were forthcoming when discussing their negative viewpoints towards patients with MUS and the challenges and difficulties faced when managing such patients. This research has identified an important gap in knowledge and training, and draws attention to the importance of educating doctors about the needs of patients with unexplained symptoms at an early and clinically-relevant stage of their career.

Participants were recruited from three hospitals in the North Thames region, and although participants had completed undergraduate training at 13 Medical Schools throughout the country, the views expressed may not represent the views of other newly-qualified doctors within the UK or elsewhere. The information gathered about management approaches was based on self-report and may not be representative of true clinical practice. Interviews varied in length due to participants’ limited availability, meaning that it was not possible to address all aspects of the topic guide in detail during a small number of the shorter interviews. As the participants volunteered to take part in the study, it is possible that they held more positive views towards MUS and had greater awareness of the topic, although negative views were actively probed for. The level of negative views expressed could be an under-representation of views held by other junior doctors. Providing participants with a book voucher could have influenced their willingness to take part, although a small number of participants refused the voucher.

## Conclusion

Current training does not equip junior doctors with the necessary knowledge and skills to effectively and confidently manage patients with MUS. Junior doctors’ educational needs must be addressed within future training programmes. Arguably one of the most relevant times for teaching about this topic is in the initial years of clinical practice, before attitudes and management styles are fully formed, but when newly-qualified doctors are starting to both meet and manage patients with unexplained symptoms on a regular basis. The findings of this study highlight a need to assist junior doctors to distinguish between feelings of uncertainty from feelings of incompetence, and provide a basis for further research to address the personal impact of working with such patients. Our results also indicate a need to target the more negative attitudes that some more senior doctors hold towards patients with unexplained symptoms, and to provide education about available management options and referral routes. Interactive training sessions involving case-based discussions, with a focus on giving meaningful explanations to patients, are recommended as a teaching method of choice.
